# MiR-29b affects the secretion of PROG and promotes the proliferation of bovine corpus luteum cells

**DOI:** 10.1371/journal.pone.0195562

**Published:** 2018-04-04

**Authors:** Ming-Qiang Xu, Hao Jiang, Li-Qun Zhang, Xu-Lei Sun, Dan Luo, Yao Fu, Yan Gao, Bao Yuan, Jia-Bao Zhang

**Affiliations:** 1 Department of Laboratory Animals, College of Animal Sciences, Jilin University, Changchun, Jilin, P.R. China; 2 Department of Obstetrics and Gynecology, The First Hospital of Jilin University, Changchun, Jilin, P.R. China; Nanjing Agricultural University, CHINA

## Abstract

The regulatory role of miRNAs has been explored in ovarian cells, and their effects on gonadal development, apoptosis, ovulation, steroid production and corpus luteum (CL) development have been revealed. In this study, we analyzed the expression of miR-29b at different stages of bovine CL development and predicted the target genes of miR-29b. We confirmed that miR-29b reduces the expression of the oxytocin receptor (OXTR), affects progesterone (PROG) secretion and regulates the function of the CL. RT-PCR showed that the expression of miR-29b was significantly higher in functional CL phases than in the regressed CL phase. Immunohistochemistry showed that OXTR was expressed in both large and small CL cells and was mainly located in the cell membrane and cytoplasm of these cells. We analyzed the expression levels of OXTR and found that transfection with a miR-29b mimic decreased OXTR expression, but transfection with the inhibitor had a limited effect on the expression of the OXTR protein. At the same time, the secretion of PROG was significantly increased in the miR-29b mimic-transfected group. We also analyzed the effect of miR-29b on the apoptosis of CL cells. Finally, we found that miR-29b could promote the proliferation of bovine CL cells. In conclusion, we found that miR-29b reduces the expression of OXTR and can promote PROG secretion and the proliferation of CL cells via OXTR.

## Introduction

The corpus luteum (CL) is a transient ovarian structure that develops rapidly after ovulation. The CL supports pregnancy via the secretion of progesterone (PROG) [1, 2]. Following ovulation and in response to the luteinizing hormone (LH) surge, the follicle undergoes luteinization, which involves the modulation of gene expression, extensive cellular proliferation, and the differentiation of granulosa and thecal cells into large and small luteal steroidogenic cells, respectively [3]. During the development of the bovine CL, its growth rate is comparable to that of the most rapidly growing tumors [4]. A large amount of the cholesterol taken up by CL cells is synthesized into PROG, androgen and estradiol, a process that is primarily controlled by oxytocin (OXT), PRL and LH.

As small non-coding RNAs that regulate signaling pathways by targeting functional genes and modulating their expression, miRNAs regulate gene expression post-transcriptionally [5, 6]. Because of the diversity of miRNA targeting [7, 8], identifying mRNA targets is critical for the discovery of tissue-specific miRNAs. Mature (21–22 nt) miRNAs can bind to the 3' untranslated region (UTR) of target mRNAs to degrade or inhibit mRNA translation [9, 10]. Several miRNAs regulate ovarian sex steroid synthesis in vitro [9]. However, there are few reports on the regulation of miRNA expression and function in the bovine CL [10].

MiR-29b is a member of the miR-29 family (which includes miR-29a, miR-29b and miR-29c). This family targets cell proliferation, cell cycle, senescence, differentiation, apoptosis, and metastasis, among other processes, making it an effective regulator of tumorigenesis and tumor progression [11, 12]. miR-29a up-regulates Wnt signaling by directly inhibiting its target genes, such as Dkk1, Kremen2 and sFRP2 [13]. The targeting of CTNNBIP1 and GSK3B by miR-29b has been shown to inhibit the Wnt pathway in murine osteoblasts and 293T cells, respectively. [14, 15] Many studies have demonstrated that miR-29b inhibits tumorigenesis, and a recent study suggested that down-regulation of miR-29a and miR-29c is closely related to the early recurrence of colorectal cancer (CRC) [16–19]. Previous studies have shown that miR-29b positively regulates osteoblast differentiation by controlling the expression of collagen and regulating inhibitory factors of the osteogenic signaling pathway in differentiated osteoblasts [15]. miR-29b suppresses angiogenesis, metastasis, and invasion by inhibiting MMP-2 expression in hepatocellular carcinoma [20]. Cortez et al. demonstrated that miR-29b targets the 3′ UTR of PDPN, inhibiting the apoptosis, proliferation and invasion of glioblastomas [21]. MiR-29b also targets the apparent genetic effects of DNA methyltransferase (DNMT3A and 3B) expression in multiple myeloma, leading to significant antitumor effects [20, 21]. However, the function and molecular regulatory mechanism of miR-29b in the development and degeneration of the bovine CL remain unclear.

The aims of this study were to analyze the expression of miR-29b at different bovine CL developmental stages, to identify target genes of miR-29b and to regulate the proliferation, apoptosis and PROG synthesis of bovine CL cells.

## Materials and methods

### Ethics statement

The protocol was approved by the Institutional Animal Care and Use Committee of Jilin University (Permit Number: 20170204).

### Collection of bovine CL samples

Ovaries with CL were collected from healthy non-pregnant Simmental cattle at a local abattoir within20 min after slaughter and transported on ice to the laboratory. Stages of CL (n = 6/stage) were classified as of early (days 1–4 after ovulation), middle (days 5–10), late (days 11–16) or regressed (days 17–21) stage as described previously [22, 23]. After the CL development phase was determined, the CL tissue was immediately isolated from the ovaries. The tissue was then processed according to the specific experimental needs: for generating paraffin-embedded tissue sections, the appropriate tissue segments were incubated in neutral formaldehyde solution; for RNA extraction, the tissue was frozen rapidly in liquid nitrogen and then stored at -80°C.

### Cell culture and transfection

CL cells were cultured in DMEM/F12 supplemented with 5% FBS, 50 units/ml penicillin and 50 ng/ml streptomycin at 37°C with 5% CO_2_. Approximately 1×104 cells/well were incubated in 6-well plates for 36 h before transfection. The cells were transfected with an miR-29b mimic, an miR-29b inhibitor, a negative control (NC) or an siRNA (RiboBio, Guangzhou, China) at a final concentration of 50 nM according to the manufacturer’s instructions.

### RNA isolation and real-time PCR

Total RNA was isolated from CL tissues and cell lines using TRIzol according to the manufacturer’s protocol (Roche Life Science, USA). The RNA concentration was determined from the A260/A280 absorbance ratio using a NanoDrop 2000 (Thermo Scientific, USA), and an A260/A280 ratio in the range of 1.8–2.0 indicated the purity of the RNA. First-strand cDNA synthesis was performed using a FastQuant RT Kit (using gDNA) according to the manufacturer’s protocol. Relative gene expression was determined via RT-PCR. The reaction mixture contained 10 μl of 2× SuperReal PreMix Plus (containing SYBR Green I), 3.2 μl of the cDNA template, 1.6 μl of the forward primer, 1.6 μl of the reverse primer and RNase-free water in a final volume of 20 μl. RT-PCR was performed using a LightCycler 480 real-time PCR system (Roche Molecular Systems, Indianapolis, USA). The reaction mixtures were incubated at 95°C for 2 min, followed by 40 cycles of 94°C for 15 s, 55°C for 15 s, and 72°C for 30 s. The real-time PCR results were normalized to U6 using the 2-ΔΔCt method. Primers were synthesized by RiboBio (Guangzhou, China). The primer sequences are shown in S1 Table.

### MiRNA target prediction

TargetScan (http://www.targetscan.org/) and MicroCosm Targets (http://www.ebi.ac.uk/enright-srv/microcosm/htdocs/targets/v5/) were used to predict miR-29b target genes.

### Dual-luciferase reporter assay

An annealed oligonucleotide pair with a putative miR-29b-3p binding site was designed and synthesized by RiboBio (Guangzhou, China) according to the bovine oxytocin receptor (OXTR) mRNA sequence in GenBank. The double-stranded product was inserted downstream of the luciferase gene in the pmiR-RB-REPORT (TM) dual-luciferase reporter vector. A double-stranded oligonucleotide containing a mutation in the miR-29b-3p seed sequence was cloned into the same region of the vector.

HEK293T cells were plated in 24-well plates, incubated for 24 h and then transfected with the miR-29b-3p mimic or NC at a concentration of 50 nM along with 600 ng of the dual-luciferase vector containing either the wild-type (WT) or mutant 3' UTR sequence. Forty-eight hours after transfection, the dual-luciferase reporter assay system (Promega) was used to detect luciferase activity.

### Immunohistochemistry

The expression of OXTR, a target gene of miR-29b in the bovine CL, was analyzed via immunohistochemistry. Paraffin-embedded sections of CL tissue at different stages were deparaffinized in a xylene solution, rehydrated in ethanol and washed with tap water. The tissue sections were then incubated in boiling buffer (0.01 M citric acid, pH 6) for 20 min for antigen recovery.

Endogenous peroxidase activity was quenched for 10 min in methanol containing 0.3% H_2_O_2_. Nonspecific binding was blocked with 5% BSA for 30 min at room temperature. The sections were then incubated overnight at 4°C with a goat anti-OXTR antibody (1:200; Abcam; ab87312). After incubation, the sections were washed with PBS and incubated with rabbit anti-goat IgG (HRP-Conjugated Anti-Goat IgG SABC Kit, Boster; SA1023) for 30 min according to the manufacturer’s instructions. Then, streptavidin-biotin-peroxidase (SABC) was added, followed by incubation for 20 min at 37°C. DAB was then added, and the sections were viewed under a microscope to determine the degree of staining and then rinsed with distilled water to terminate the reaction. The sections were counterstained with Mayer's hematoxylin and finally dehydrated and sealed.

### Western blotting

Proteins were extracted from samples of CL tissue at the four different developmental stages using a lysis buffer containing protease and phosphatase inhibitors (Sangon, Shanghai, China) according to the manufacturer’s instructions. Cell debris was removed by centrifugation at 4°C for 30 min at 17000 x g. A Bradford Protein Assay Kit (Sangon) and a Synergy Multi-Mode Microplate Reader were used to measure protein concentrations by determining the absorbance at 595 nm. Then, 40 μg of total protein was fractionated on a 12% SDS-PAGE gel and transferred to a polyvinylidene fluoride membrane for Western blot analysis. The membrane was then blocked with 5% BSA at room temperature for 1 h and probed with a goat polyclonal antibody (1:500; Abcam; ab87312) overnight at 4°C. The membrane was washed 2–5 times and then incubated with horseradish peroxidase-conjugated rabbit anti-goat IgG at room temperature for 60 min (Santa Cruz). The protein bands were visualized using SuperSignal, West, and Pico chemiluminescence substrates (Pierce, Rockford, IL, USA).

### PROG detection

We collected 50 μl of supernatant from CL cells 36 h after transfection. The PROG levels under different experimental conditions were measured using a Bovine PROG ELISA Kit according to the manufacturer’s instructions (Haling Biotech Co., LTD., Shanghai, China).

### CCK-8 analysis

Thirty-six hours after transfection, 1 x 104 cells in 100 μl of medium were seeded into each well of a 96-well plate, with three wells per group. The plates were incubated for 3.5 h. Then, the optical density in each well was measured at 450 nm under a flat reading device calibrated to a blank well. For each group, the average of three wells was obtained to generate the proliferation curve.

### Flow cytometric analysis of CL cells apoptosis

The following apoptosis detection steps were performed as described in the FITC Annexin-V Apoptosis Detection Kit I (BD, USA). First, cells and cell debris were digested with trypsin without EDTA 36 h after cell transfection. Next, the cells were washed with PBS and centrifuged at 500 x g 2 to 3 times at 5°C (5 min each). Then, 100 μl of PBS, 5 μl of FITC solution and 5 μl of propidium iodide (20 μg/ml) were added to each centrifuge tube, and the samples were incubated at 25°C for 15 min in the dark. Finally, the samples were measured within 2 h using flow cytometry (BD, USA). Apoptotic cells and dead cells were stained with propidium iodide and FITC.

### Statistical analysis

All experimental data are shown as the average values ± standard deviation (SD). SPSS 11.5 was used for the statistical analysis. Statistically significant differences were examined using Student’s t-test or one-way analysis of variance (ANOVA). Spearman rank correlation coefficients were used for correlation analyses. A probability of less than 5% (P < 0.05) was considered significant.

## Results

### Expression of miR-29b and OXTR at different stages of bovine CL development

**Fig 1:**
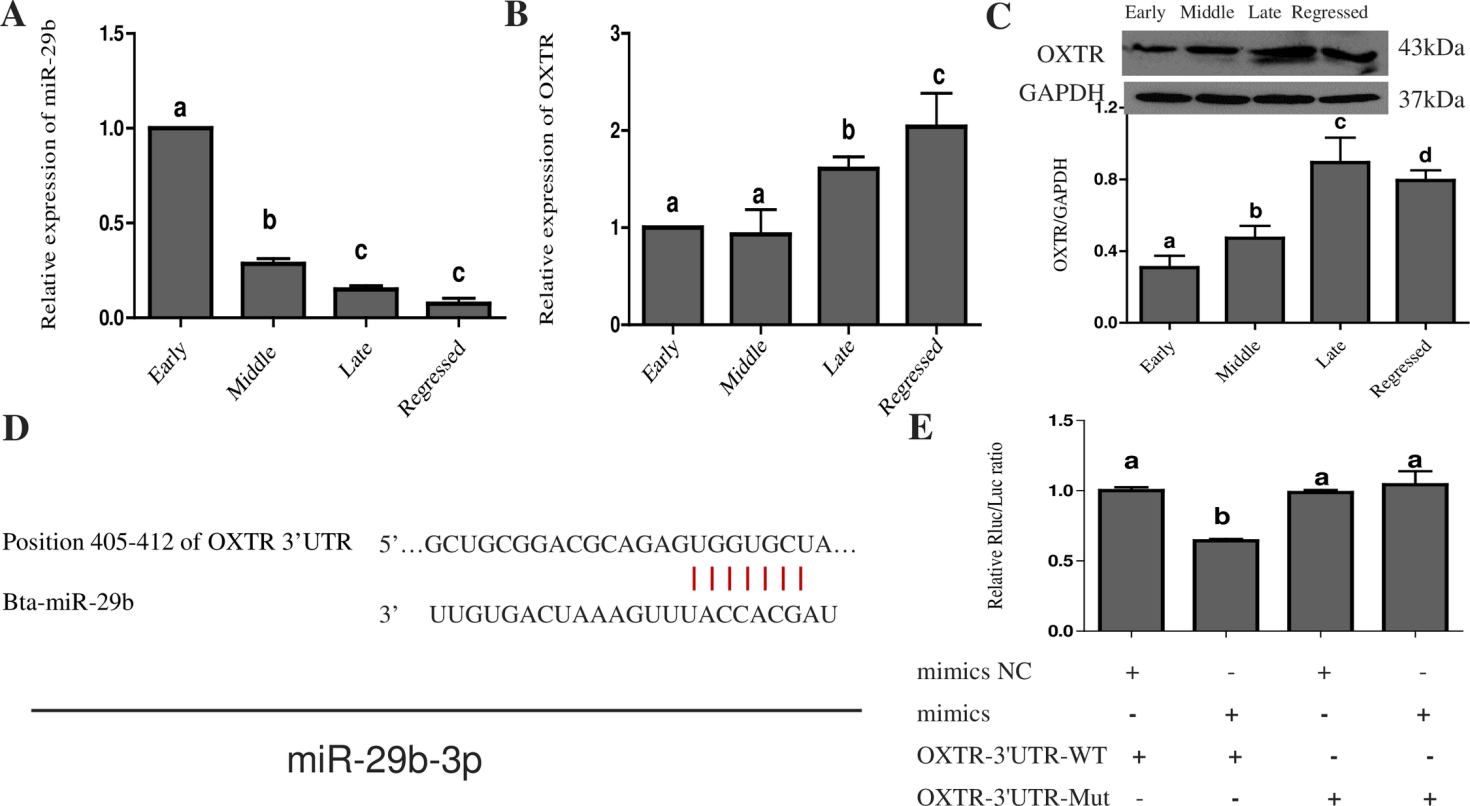
Expression of miR-29b and OXTR at different stages of bovine CL development and validation of its target genes. (A) Relative expression of miR-29b. (B) The relative expression of the OXTR gene at the four CL developmental stages was determined using RT-PCR. The Y-axis shows the relative mRNA expression levels normalized to the expression level of U6. Values are presented as the means ± standard deviation from three independent experiments. Superscript letters indicate significant differences (P < 0.05). (C) Western blot analysis of the OXTR protein and relative quantification of OXTR protein expression at different CL developmental stages. Total protein for each stage was pooled from three CL samples. The Western blotting data are representative data from biological replicates. GAPDH was used as an internal control. (D) Sequence alignment of miR-29b-3p with the 3' UTR of OXTR. (E) Relative luciferase activity of the pmiR-OXTR-3'UTR-WT and pmiR-OXTR-3'UTR-MUT vectors in 293T cells co-transfected with a miR-29b mimic or a negative control. The relative luciferase activity was measured 48 h after transfection and was normalized to that of cells transfected with the pmiR-RB-REPORTTM vector. The normalized luciferase activity of the control was set to 1, and the data are presented as the means ± standard deviation from at least three independent experiments. Statistical significance was determined by one-way ANOVA. The same letters indicate that the difference is not significant, and different letters (a, b and c) indicate that the difference is significant (P < 0.05). U6 was used as the standard control.

To investigate the role of miR-29b in bovine CL, we analyzed the expression levels of miR-29b at different stages of CL development. The RT-PCR results revealed that miR-29b showed the highest expression in the early CL and then gradually decreased, with the lowest expression observed in the regressed CL; the difference between the late and regressed CL was significant (Fig 1A). OXTR is a G protein-coupled seven transmembrane domain receptor found in many tissues, including the testis, ovary, thymus, and brain. In the mammalian ovary, OXT influences steroidogenesis, luteinization, ovulation, and luteal regression. The OXT-OXTR system has been extensively studied in bovines and humans. Therefore, we also analyzed the expression of OXTR in the bovine CL at different stages to verify the potential relationship between miR-29b and OXTR. The results of RT-PCR and Western blot showed that the expression of OXTR was low in the early and middle CL (Fig 1B and 1C); these results indicate that OXTR is one of the potential target genes of miR-29b in the bovine CL.

### OXTR is a target gene of miR-29b in bovine CL

To examine whether OXTR silencing is mediated by a specific, direct interaction between miR-29b-3p and the OXTR target site, we predicted the target position of miR-29b-3p in the OXTR 3' UTR using the TargetScan program (Fig 1D). We then constructed the pmir-OXTR-3'UTR and pmir-mutant-OXTR-3'UTR vectors and co-transfected pmir-OXTR-3'UTR into HEK293T cells with a miR-29b-3p mimic, which led to an approximately 30% reduction in luciferase activity; however, co-transfection of the miR-29b-3p mimic and the pmir-mutant-OXTR-3'UTR vector yielded no significant changes in luciferase activity (Fig 1E). Thus, the results suggested that miR-29b-3p can regulate the expression of OXTR.

### Localization analysis of OXTR at different CL developmental stages

**Fig 2:**
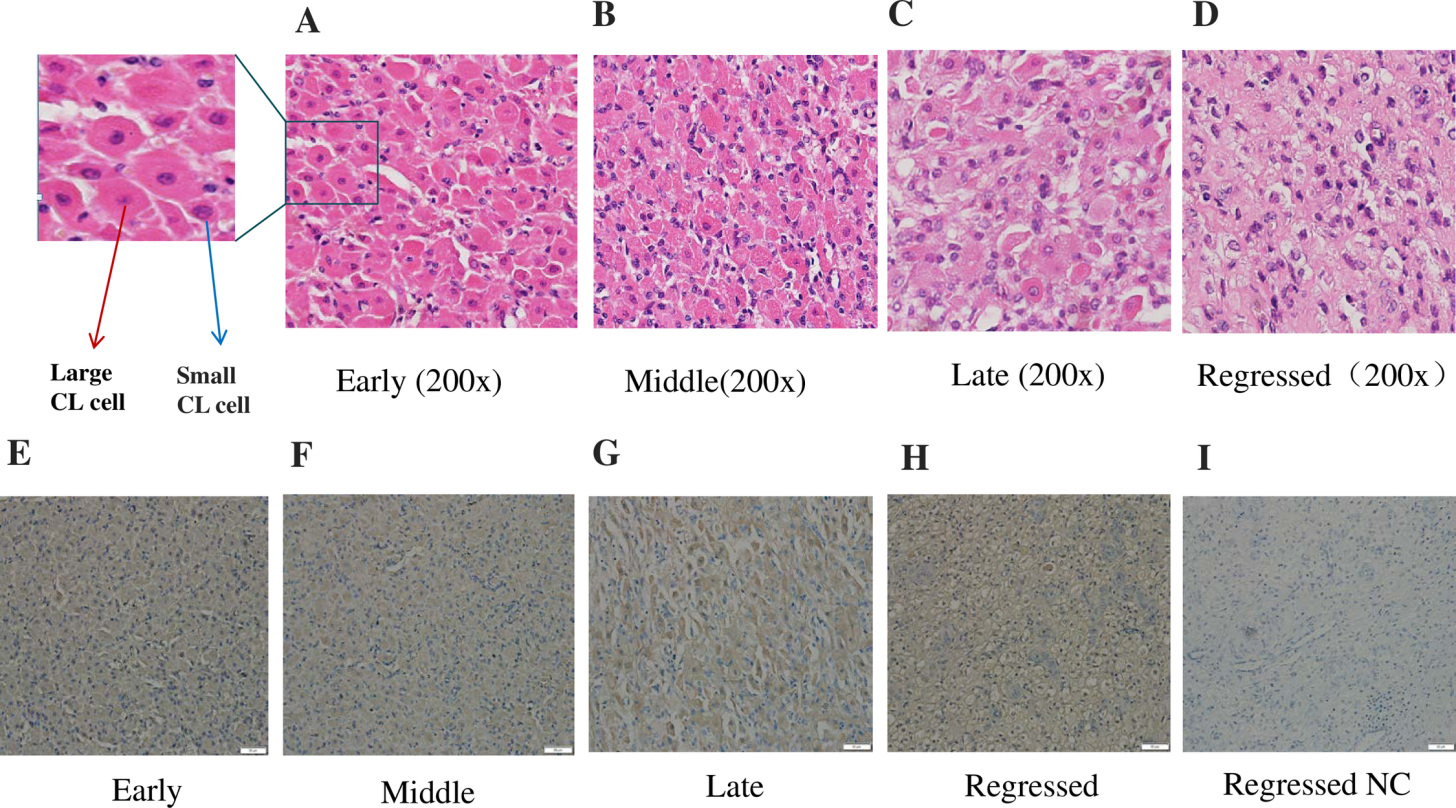
Localization of OXTR at different CL developmental stages. (A), (B), (C) and (D) HE staining at different periods of bovine CL development. Red arrows indicate large CL cells, and blue arrows indicate small CL cells. Original magnification 200×. (E), (F) (G), (H) and (I) Immunohistochemical analysis of OXTR localization during different stages of bovine CL development. Scale bar, 50 μm.

To further investigate the function of OXTR in the CL, we analyzed the localization of OXTR at different CL stages. HE staining showed that the histological structure and cell composition of the CL differed significantly at different CL developmental stages (Fig 2A, 2B, 2C and 2D), while immunohistochemistry showed that OXTR was expressed in the large and small CL cells at different developmental stages and was mainly located in the cytoplasm and cell membrane (Fig 2E, 2F, 2G, 2H and 2I).

### OXTR is regulated by miR-29b in CL cells

**Fig 3:**
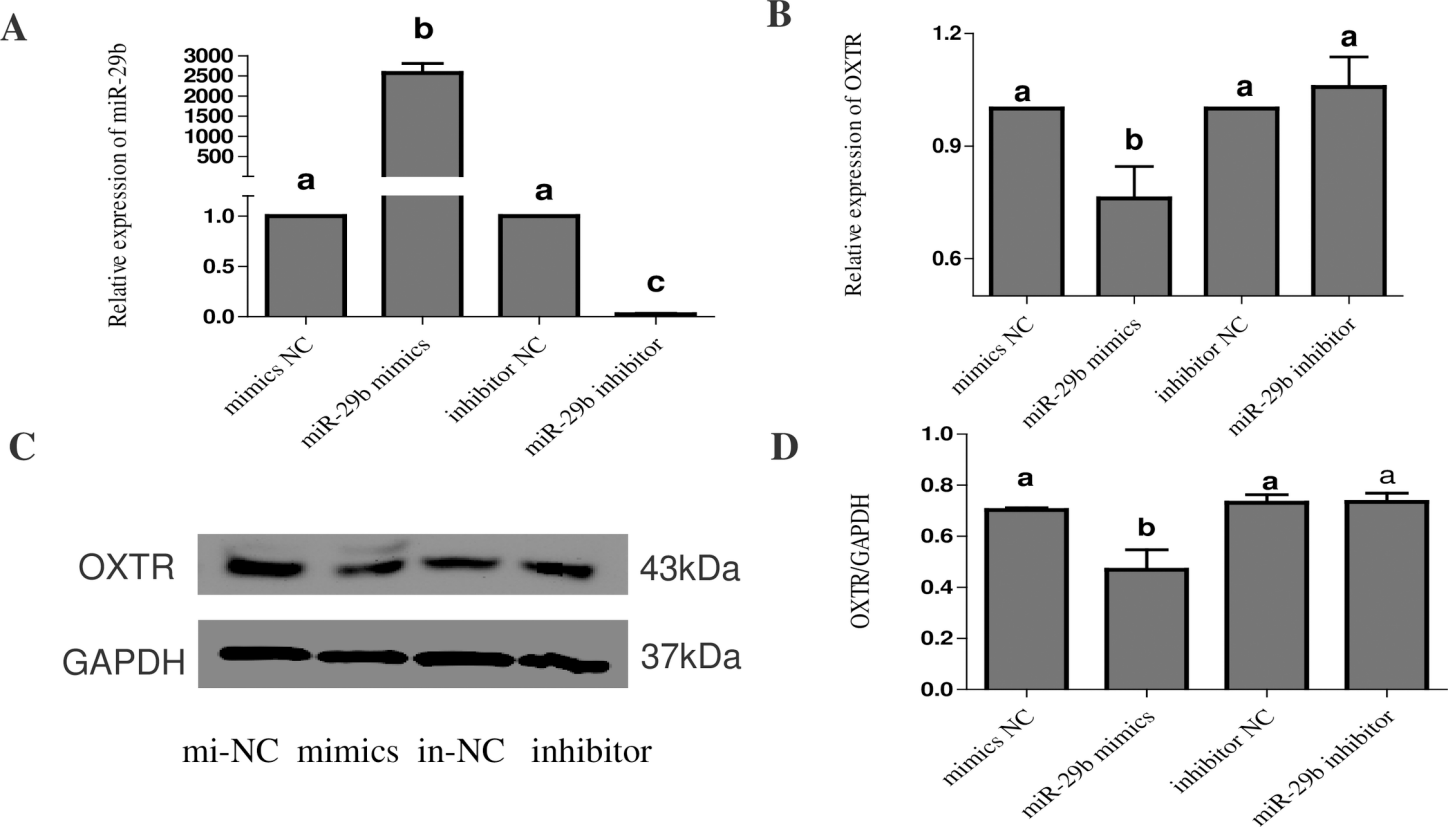
Transfection efficiency of miR-29b and its regulation of the target gene OXTR. Bovine CL cells were transfected with negative control, miR-29b mimic or miR-29b inhibitor. (A) Relative expression of miR-29b. (B) Relative expression of OXTR. (C) Western blot analysis of OXTR protein expression. (D) Relative quantification of OXTR protein expression after transfection of miR-29b mimic and inhibitor. OXTR expression was also analyzed using quantitative RT-PCR with GAPDH as an internal control. All data are presented as the means ± standard deviation from at least three independent experiments. Statistical significance was determined via one-way ANOVA. The same letters indicate that the difference is not significant, and different letters (a, b and c) indicate that the difference is significant (P < 0.05).

To further study the regulatory effect of miR-29b on OXTR, we transfected CL cells with a miR-29b mimic, miR-29b inhibitor or NC. The transfection efficiency was measured using RT-PCR. Transfection with the miR-29b mimic significantly increased the expression level of miR-29b, and transfection with the miR-29b inhibitor significantly decreased the expression level of miR-29b, indicating that the transfection was successful (Fig 3A). At the same time, we analyzed the expression level of OXTR and found that transfection with the mimic decreased OXTR expression, but transfection with the inhibitor had little effect on the expression of the OXTR protein (Fig 3B, 3C and 3D). These results demonstrated that miR-29b could reduce the expression of OXTR in bovine CL cells, indicating that OXTR is a miR-29b target gene.

### miR-29b influences PROG secretion in bovine CL cells via OXTR

**Fig 4:**
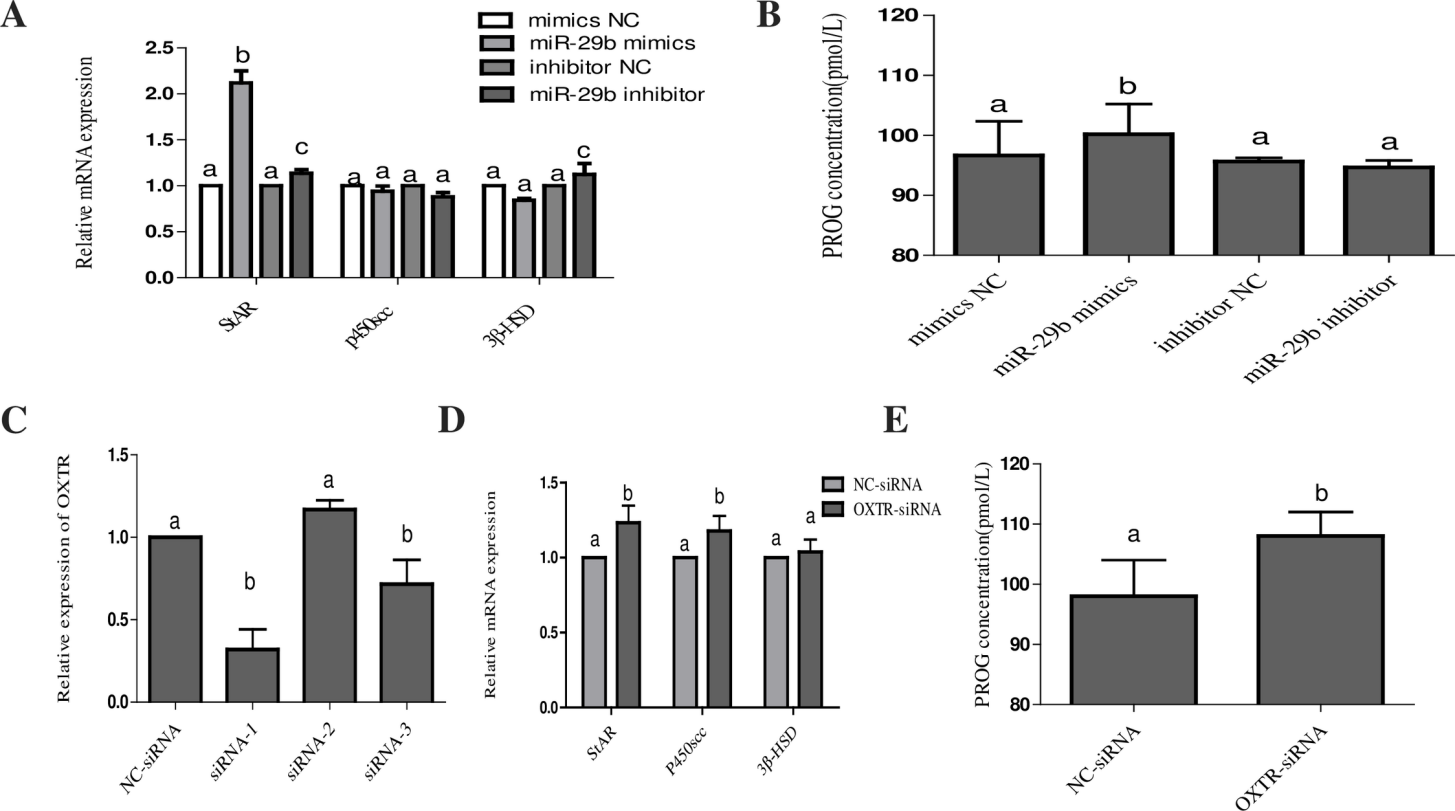
Effects of miR-29b on key genes involved in progesterone synthesis and progesterone secretion. (A) and (D) The expression of key genes involved in PROG synthesis in CL cells was detected by RT-PCR. (B) and (E) PROG concentrations (pmol/L). Bovine CL cells were transfected with a mimic NC, miR-29b mimic, inhibitor NC, miR-29b inhibitor, NC-siRNA or OXTR-siRNA. (C) Effects of different siRNAs on OXTR. Cell supernatants (50 μl) were collected 48 h after transfection and examined using a Bovine PROG ELISA Kit. All data are presented as the means ± standard deviation from at least three independent experiments. Statistical significance was determined via one-way ANOVA. The same letters indicate that the difference is not significant, and different letters (a, b and c) indicate that the difference is significant (P < 0.05).

We transfected bovine CL cells with the mimic NC, miR-29b mimic, inhibitor NC, miR-29b inhibitor, NC-siRNA or OXTR-siRNA at 50 nM and subsequently incubated the cells for 36 h. During the synthesis of steroid hormones, cholesterol is transported into the outer mitochondrial membrane and localizes to the intermembrane space; this step is also the rate-limiting step in the steroid synthesis pathway. Steroidogenic acute regulatory protein (StAR), 3β-hydroxysteroid dehydrogenase (3β-HSD) and P450 cholesterol side-chain cleavage enzyme (P450scc) are three very important enzymes in this process. To investigate the effect of miR-29b on PROG, we used RT-PCR to detect the expression of key genes involved in PROG synthesis at the mRNA level. StAR expression was significantly increased after overexpression of miR-29b in CL cells. However, there was no significant change in the expression of 3β-HSD or P450scc (Fig 4A). Then, we measured the concentrations of PROG in these 4 groups. PROG secretion was significantly increased in the miR-29b mimic group but showed almost no change in the miR-29b inhibitor group (Fig 4B). In addition, OXTR-siRNA was designed to demonstrate that miR-29b affects PROG synthesis via OXTR in the bovine CL. We designed three siRNAs, and the results showed that the knockout efficiency of sRNA-1 was the best. Therefore, we chose siRNA-1 (OXTR-siRNA) for follow-up experiments. We found that the expression of StAR and P450scc and the secretion of PROG were significantly increased in the OXTR-siRNA group, which indicates that miR-29b affects PROG secretion in the bovine CL by regulating the expression of OXTR (Fig 4C, 4D and 4E).

### Effects of miR-29b on the proliferation and apoptosis of bovine CL cells

**Fig 5:**
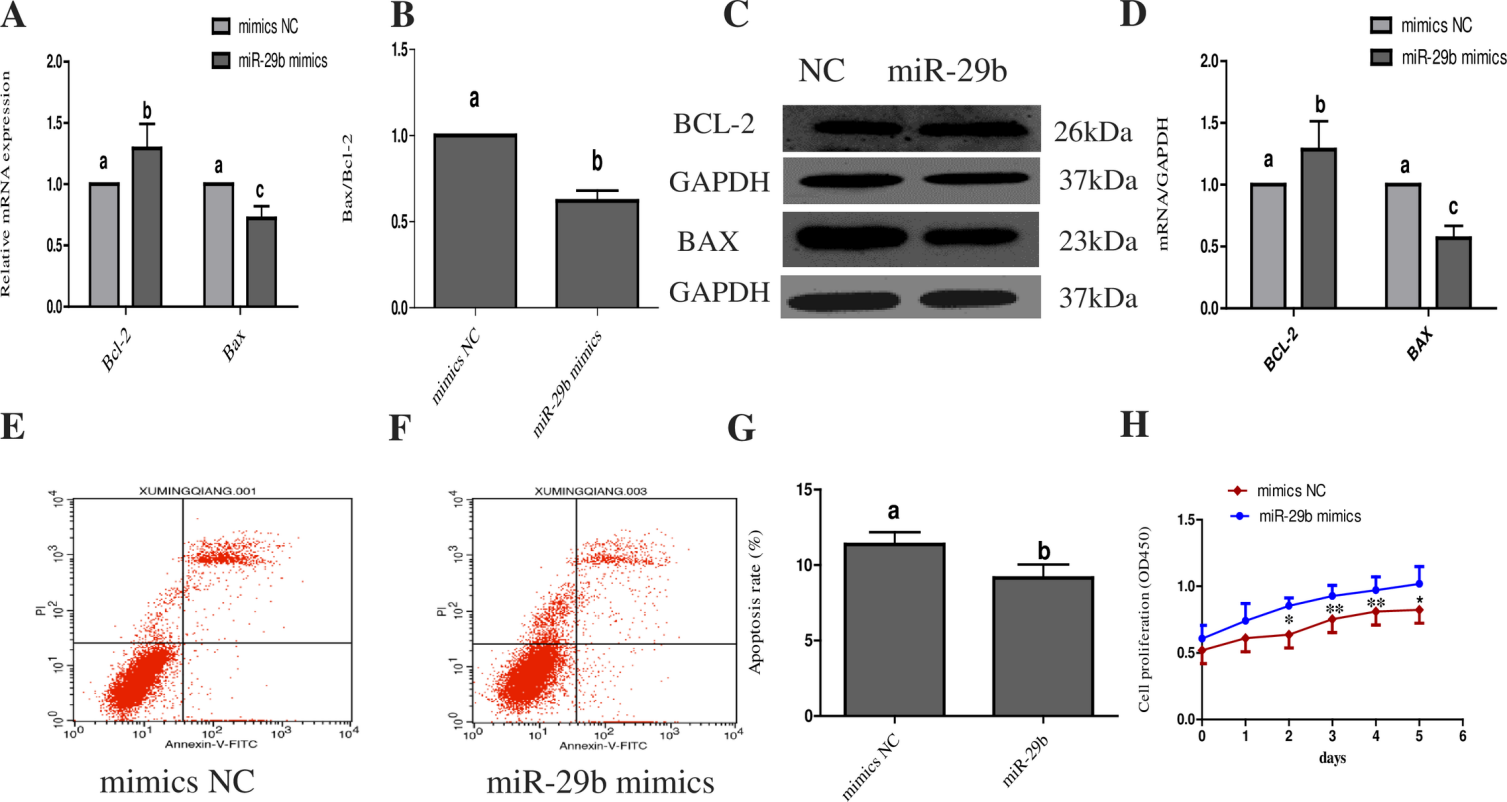
Effects of miR-29b on the proliferation and apoptosis of CL cells. (A) Bcl-2 was up-regulated and Bax was down-regulated in CL cells transfected with a miR-29b mimic. (B) The ratio of Bax to Bcl-2 was reduced in CL cells transfected with miR-29b mimic. (C) Bcl-2 and Bax protein expression in CL cells. (D) Relative gray value analysis of Bax and Bcl-2 protein expression. (E) and (F) Flow cytometry was performed to observe the apoptosis of CL cells via staining with Annexin-V-FLUOS/PI. (G) Apoptosis rates of CL cells after transfection with miR-29b mimic or NC. (H) Analysis of the viability of CL cells using the CCK-8 method. All data are presented as the means ± standard deviation from at least three independent experiments. Statistical significance was determined via one-way ANOVA. The same letters indicate that the difference is not significant, and different letters (a, b and c) indicate that the difference is significant. P < 0.05 was considered significant. * P < 0.05, ** P < 0.01.

The expression levels of Bcl-2 and Bax in CL cells were detected via Western blotting and RT-PCR after transfection with the miR-29b mimic or NC for 36 h. In CL cells transfected with the miR-29b mimic, the expression of Bcl-2 was significantly increased compared with cells transfected with the NC, and the expression level of Bax was significantly decreased after miR-29b overexpression (Fig 5A, 5B, 5C and 5D). Flow cytometry was used to analyze the apoptosis of bovine CL cells, and the apoptosis rate of the NC group was significantly higher than that of the miR-29b group (Fig 5E, 5F and 5G). This finding suggests that miR-29b may inhibit the apoptosis of CL cells, so we used the CCK-8 method to verify whether miR-29b affects the proliferation of CL cells. Over-expression of miR-29b significantly enhanced the proliferation ability of CL cells, and the difference between the miR-29b group and the NC group was statistically significant (Fig 5H).

## Discussion

The CL is a dynamic endocrine organ in the ovary. The CL develops from an ovarian follicle and induces endometrial cell differentiation; in the absence of fertilization, the CL degrades, and the formation and degradation of the CL regulate hormone levels in the body [24, 25]. Therefore, the normal physiological processes of CL cells play an important role in the normal reproductive regulation of the body. In addition, studies have shown that miRNAs play important roles in luteal development and maintenance [26]. miR-29a, -29b1, -29b2 and -29c are members of the miR-29b family. miR-29a and -29b1 are located in an intron of human chromosome 7, while miR-29b2 and -29c are located on chromosome 1. These miRNAs are highly conserved among humans, rats and mice [27]. miR-29b1 and -29b2 are collectively referred to as miR-29b because they have the same mature sequence. Abnormal expression of miR-29b family members has been found in various human cancers, including breast cancer and non-small cell lung cancer [28–30]. miR-29b is up-regulated during mESC differentiation, consistent with its role in the induction of pluripotent stem cells [31]. Although miR-29b plays an important role in cellular metabolism, its role in CL development is unknown. In this study, we found that the level of miR-29b gradually decreased from the early to regressed stage of CL development, which may indicate that miR-29b promotes CL development.

To study the role of miR-29b in the CL, we verified its target genes and found that miR-29b acts directly on OXTR. OXTR is a G protein-coupled seven transmembrane domain receptor found in many tissues, including the testis, ovary, thymus, and brain [32]. In the mammalian ovary, OXT influences steroidogenesis, luteinization, ovulation, and luteal regression [33]. The OXT-OXTR system has been extensively studied in cattle and humans.

In the presence of hCG, the downstream effect of OXT binding to OXTR on granulocytes is due to an increase in PROG production caused by luteinization [17]. OXTR has been previously detected in the CL of primates and in preovulatory follicles [34, 35]. Our research also provides some surprising insights into other cellular sites expressing OXTR in the bovine CL. Combined immunohistochemical and RNA analyses revealed that OXTR is not only expressed during the four stages of CL development but is also expressed at each stage by the large CL cells and small CL cells. Since down-regulation of OXTR in the ovaries and up-regulation of OXTR in the uterus have been proposed to induce the onset of parturition, elevated expression of OXTR in the ovaries of Mamld1-KO mice may be associated with delayed parturition [36]. RT-PCR and Western blotting showed that the expression of OXTR in the functional CL (middle stage) was significantly lower than that in the degenerative CL (regressed stage), indicating that OXTR may regulate the processes of CL formation and degeneration. However, the underlying mechanism requires further investigation.

Bcl-2 family members are important regulators of the mitochondrial apoptotic pathway [37]. Bcl-2 family members can be divided into pro-apoptotic and anti-apoptotic proteins based on the number of Bcl-2-homologous domains and their biological effects on apoptosis [38]. Bcl-2 and Bax are two key proteins that regulate cell survival and apoptosis. The anti-apoptotic protein Bcl-2 binds to the proapoptotic protein Bax in the mitochondrial outer membrane, preventing Bax from forming homodimers and inhibiting apoptosis. When cells are stimulated by apoptotic signals, the number of Bax molecules is increased via Bax expression up-regulation or Bcl-2 expression down-regulation. This event disrupts the Bax/Bcl-2 balance and allows free Bax to form homodimers on the mitochondrial membrane, leading to changes in mitochondrial membrane permeability [38]. Therefore, we used RT-PCR to examine the changes in Bcl-2 and Bax levels after the expression of miR-29b in CL cells. We found that overexpression of miR-29b could up-regulate Bcl-2 and down-regulate Bax mRNA, suggesting that miR-29b may promote the proliferation of CL cells. CCK-8 experiments further confirmed this conclusion.

The main physiological function of the CL is the synthesis of steroid hormones such as PROG, which plays an important role in regulating changes in the endometrium to accommodate fertilized egg implantation and early pregnancy maintenance [39]. Cholesterol is a precursor in the synthesis of steroid hormones. Under normal circumstances, most of the cholesterol synthesized by the liver is transported in the form of lipoproteins through the blood circulation to tissues or organs that secrete steroid hormones, which reach cells in different ways [40]. During the synthesis of steroid hormones, cholesterol is transported into the outer mitochondrial membrane and localizes to the intermembrane space; this step is also the rate-limiting step in the steroid synthesis pathway. StAR has a very important effect on the transport of cholesterol [41]. Once the cholesterol is transported into the mitochondrial matrix, P450scc converts cholesterol into pregnenolone, which is free to diffuse into the cytoplasm. Then, 3β-hydroxysteroid dehydrogenase (3β-HSD) in the smooth endoplasmic reticulum converts pregnenolone into biologically active PROG. The synthesized PROG is then secreted from CL cells and exerts its physiological effects. In this study, the PROG secretion of CL cells increased after treatment with miR-29b mimics. The amount of PROG secretion from CL cells was significantly increased after OXTR knockdown with siRNA, indicating that miR-29b may promote PROG secretion by inhibiting the expression of OXTR.

Taken together, our results indicate that miR-29b can significantly enhance PROG secretion in CL cells by both promoting the proliferation of CL cells, resulting in an increased number of cells, and increasing the expression of the key gene in steroid hormone synthesis, StAR, eventually leading to increased secretion of PROG.

## Supporting information

S1 Table Primers for reverse transcription and quantitative real-time PCR. (DOCX)

S2 Table Raw data for Figs 1–5. (XLSX)
