# Supplementary material for: MiR-29b affects the secretion of PROG and promotes the proliferation of bovine corpus luteum cells
Source: PLoS One. 2018 Apr 4;13(4):e0195562. doi: 10.1371/journal.pone.0195562 (PMC5884578; doi:10.1371/journal.pone.0195562)
Supplement: S1 Table — (DOCX) [file pone.0195562.s001.docx]

S1 Table. Primers for reverse transcription and quantitative real-time PCR.

| Primers | Sequences (From 5′to 3′) | | |
| --- | --- | --- | --- |
| U6-F | | | TCGCTTCGGCAGCACATATAC |
| U6-R | | | GCGTGTCATCCTTGCGCAG |
| U6-RT | | | CGCTTCACGAATTTGCGTGTC |
| miR-29b-F | | | GCGTAGCACCATTTGAAATC |
| miR-29b-R | | | CAGTGCGTGTCGTGGAGT |
| miR-29b-RT | | | GTCGTATCCAGTGCGTGTCGTGGAGTCGGCAATTGCACTGGATACGACAACACT |
| OXTR-F | | | TCAGCCAACGTCAAGCTCATC |
| OXTR-R | | | TCCACATCTGCACGAAAAAG |
| Bax-F | | | TTCTGACGGCAACTTCAACTG |
| Bax-R | | | GGTGTCCCAAAGTAGGAGAGG |
| Bcl-2-F | | | GTGTGTGGAGAGCGTCAACC |
| Bcl-2-R | | | GAGACAGCCAGGAGAAATCAAAC |
| StAR-F | | | CAGCAGAAGGGTGTCATCAG |
| StAR-R | | | ATCCCTTGAGGTCAATGCTG |
| P450scc-F | | | ATCATTCACCCTGAAGACGTG |
| P450scc-R | | | TCTTAAACAGGACTCCAATGGG |
| 3β-HSD-F | | | ACACCGCCTCTGTCATTG |
| 3β-HSD-R | | | TGGTGCTGGTGTGGATAAAG |
| GAPDH-F | | | ACATACTCAGCACCAGCATCAC |
| GAPDH-R | | | ATTCTGGCAAAGTGGACATCG |
